# A retrospective study of cochlear implant outcomes in children with residual hearing

**DOI:** 10.1186/1472-6815-6-7

**Published:** 2006-04-19

**Authors:** Elizabeth Fitzpatrick, Rosemary McCrae, David Schramm

**Affiliations:** 1 Institute of Population Health, University of Ottawa, Ottawa, Canada; 2 Children's Hospital of Eastern Ontario Research Institute, Ottawa, Canada; 3 Children's Hospital of Eastern Ontario, Ottawa, Canada; 4 Department of Otolaryngology, University of Ottawa, Ottawa, Canada

## Abstract

**Background:**

There has been increasing demand for the cochlear implantation of children who demonstrate some auditory capacity with conventional hearing aids. The purpose of this study was to examine speech recognition outcomes in a group of children who were regarded as borderline candidates for cochlear implantation as their residual hearing and/or auditory functioning levels exceeded typical audiologic candidacy criteria.

**Methods:**

A retrospective chart review was undertaken at one Canadian cochlear implant centre to identify children implanted at age 4 or older with a pure-tone-average of 90 dB or better and speech recognition of 30% or greater. Pre-implant and post-implant open-set word and sentence test scores were analyzed.

**Results:**

Eleven children of 195 paediatric cochlear implant recipients met the inclusion criteria for this study. Speech recognition results for the10 English-speaking children indicated significant gains in both open-set word and sentence understanding within the first 6 to 12 months of implant use. Seven of 9 children achieved 80% open-set sentence recognition within 12 months post-surgery.

**Conclusion:**

Children with several years of experience using conventional amplification demonstrated rapid progress in auditory skills following cochlear implantation. These findings suggest that cochlear implantation may be an appropriate intervention for selected children with severe hearing losses and/or auditory capacity outside current candidacy criteria.

## Background

Cochlear implants have considerably improved speech and language outcomes in children with bilateral severe to profound hearing loss [1,2]. Cochlear implantation is typically offered to individuals who receive limited benefit from conventional stimulation with well-fitted hearing aids. The definition of "limited benefit" for children has changed appreciably in the past 15 years. Early criteria for paediatric cochlear implantation restricted the procedure to children with profound hearing loss who derived essentially no benefit from conventional hearing aids. However, as cochlear implant technology progressed and documented outcomes exceeded early expectations, the audiologic boundaries of candidacy broadened to include children with more residual hearing. Current paediatric audiologic criteria include a sensorineural hearing loss of 90 dB HL or greater and up to 30% or less open-set word speech recognition. However, there appears to be considerable variation in clinical practice regarding the implantation of children with hearing thresholds and/or functional auditory abilities outside these selection criteria. Paediatric cochlear implantation seems now to have arrived at a point of clinical equipoise where there is considerable uncertainty about the boundaries for audiologic criteria in the application of this technology.

The effectiveness of cochlear implantation for children with bilateral severe to profound hearing loss is well documented [1,3]. However, clinical decisions regarding selection criteria for children with pre-implant residual hearing are complicated by the fact that a wide range of performance has been documented after implantation [4]. A recent systematic review by our research group which analyzed published results of prospective studies of children implanted during the preschool years, found that average open-set speech recognition results ranged between 40% and 70% after 4 -5 years of implant experience (Fitzpatrick et al., unpublished data). Furthermore, the boundary beyond which a cochlear implant offers greater benefit than conventional amplification may also be influenced by other child and family factors such as age of implantation, family involvement, post-implant rehabilitation and educational mode [1,4-6].

Since cochlear implants first received U.S. Food and Drug Administration (FDA) approval, audiologic selection criteria have been expanded for both adults and paediatric patients. For adults, selection criteria have changed from a profound hearing loss and limited open-set speech recognition in the early 1990s to a 70 dB hearing loss and up to 50% open-set sentence speech perception [7]. Although the broadening of the selection criteria to a 70 dB pure-tone-average hearing loss occurred in 1995 for the adult population, the criteria for children remain a pure-tone- average of less than or equal to 90 dB HL.

Several studies have investigated whether cochlear implantation is beneficial for marginal hearing aid users. In several adult outcome studies, residual hearing and better speech recognition scores before implantation have appeared to be determinants of cochlear implant benefit [8]. Overall, there is empirical support for the implantation of adults with acquired deafness and significant open-set speech perception [7].

Our recent systematic review did not identify any controlled intervention trials comparing the outcomes for groups of "borderline" children who use hearing aids with those who use cochlear implants. Several investigators have compared the auditory capacity of children with cochlear implants to children with hearing aids by using the functional equivalent average hearing level concept. In 1994, Boothroyd and Eran [9] reported that the best cochlear implant users were functioning similar to children with a hearing loss of 70 to 89 dB HL. Several other authors have subsequently explored the equivalent hearing loss concept through a variety of procedures [10-14]. These studies report average functional hearing levels for implanted children ranging from 77 to100 dB HL. The best performers after implantation demonstrate functional abilities comparable to children with hearing loss of 70 to 80 dB HL. This wide range of performance explains the difficulty in drawing conclusions about the benefits of cochlear implantation for any individual child.

Early studies suggested that children with a hearing loss greater than 100 dB HL performed better with a cochlear implant while children with a hearing loss of 90–100 dB HL and early auditory instruction showed results similar to that of hearing aid users. In 1997, Geers [15] cautioned against implanting children with hearing levels less than 90 dB. Recent publications suggest that in some centres, paediatric implantation criteria have broadened to include children whose hearing thresholds and/or pre-implant auditory capacity exceed typical selection criteria [16-18]. These investigators advocate implantation of "select" patients with less severe hearing loss who have previously developed auditory skills with hearing aids. However, identifying these particular patients remains a challenging process supported by little empirical evidence.

The degree of hearing loss or auditory functioning that determines cochlear implant candidacy varies across clinical programs. In some cases, both parents and educators have become strong advocates for implanting children whose auditory skills are outside the normally accepted criteria. This creates a dilemma for the cochlear implant team – how much hearing is too much for a cochlear implant? To address this issue, it is useful to document outcomes after implantation on children who already derive significant benefit from conventional acoustic stimulation. This is particularly important in view of the relatively small number of "borderline" children who have received cochlear implants to date and the apparent increasing demand for this intervention [12]. The purpose of this study was to build on previous reports by examining the improvement in auditory functioning after cochlear implantation for children who were considered "borderline" candidates at our centre.

## Methods

### Participants

In this retrospective study, participants were identified through a review of the clinical records of 195 children implanted since 1993 at a paediatric hospital in Canada. This study was approved by the Research Ethics Board at the Children's Hospital of Eastern Ontario.

The study included children classified as "borderline" candidates according to one of the following criteria: 1) bilateral sensorineural hearing loss with a pure-tone-average (500, 1000, 2000 Hz) of < 90 dB, 2) significant pre-implant open-set speech perception results (≥ 20% on monosyllabic tests or ≥ 50% on sentence tests). Only children who were implanted at age ≥ 4 years were included in this study to permit speech recognition data to be extracted as an outcome measure. Children with a clinical diagnosis of auditory neuropathy were excluded due to the different audiologic profile which has been reported for this population. This includes great variability in behavioural audiometric thresholds and speech understanding skills that differ from children with comparable degrees of sensorineural hearing loss [19].

Eleven children met the audiologic inclusion criteria for this study. Ten of the 11 children meeting the audiologic criteria received rehabilitation services in English. One patient who spoke French was excluded from the study as this child was not administered the same test protocol. A summary of the characteristics of the 10 English-speaking participants is provided in Table 1.

**Table 1:** Demographic Data for 10 Implanted Children with Residual Hearing

Participant	Etiology	Clinical Course	Age at Diagnosis (years)	Age Hearing Aids Fitted (years)	Age Profound (years)	Age at CI Activation (years)	Duration of CI Use (months)	PTA CI side (dB HL)	PTA non-CI side (dB HL)
**1**	NICU	Progressive	0.62	1.06	5.62	6.05	49	98.3	95.0
**2**	Unknown	Progressive	2.53	2.69	4.21	4.47	43	108.3	78.0
**3**	Unknown	Congenital	2.48	2.53	2.49	14.61	65	96.6	96.6
**4**	Meningitis	Progressive	0.26	0.67	3.74	7.07	46	96.6	78.3
**5**	Unknown	Progressive	2.48	2.53	7.95	8.80	42	93.3	93.3
**6**	NICU	Progressive	0.73	0.80	4.63	6.93	40	101.6	83.3
**7**	NICU	Progressive	0.70	0.86	3.29	4.09	12	90.0	90.0
**8**	Familial	Progressive	3.28	3.34	10.58	12.11	25	93.3	96.6
**9**	Familial	Progressive	0.09	0.21	3.68	6.05	15	100.0	98.0
**10**	Unknown	Progressive	4.75	4.89	6.44	6.71	18	96.7	101.6

KEY: CI: cochlear implant; PTA: pure-tone-average; HL: hearing level; NICU: neonatal intensive care unit;

The majority of children were implanted after several years of consistent hearing aid use. The mean age at implant activation for the 10 English-speaking children with speech recognition results was 7.7 years (range 4.1 to 14.6 years). Pre-implant hearing aid experience ranged from 1.8 to 12.1 years (mean 5.7 years). The duration of implant use ranged between 1 and 5 years. Nine of the 10 children had fluctuating or progressive hearing loss. The mean pure-tone-average and range for the last pre-implant audiogram were: implanted ear – 97.5 dB HL (range 90.0 to 108.3); non-implanted ear – 91.1 dB HL (range 78.0 to 101.6). At the time of data collection, none of the children presented with a documented disability in addition to hearing loss that would interfere with the typical acquisition of spoken language. All participants were enrolled in an intensive auditory-verbal therapy program focused on developing auditory and oral communication skills prior to implantation. The decision to perform cochlear implantation was made in close collaboration with the parents, the rehabilitation members of the clinical implant team as well as the educators in the school system where applicable. All children continued to receive auditory-verbal intervention after surgery either at the implant centre and/or in the school system.

No child in this study continued to wear a hearing aid in the contralateral ear for an extensive period post-surgery. At the time this group of children was implanted, the practice of the implant centre was to provide children the option of using bimodal stimulation (cochlear implant and hearing aid) based on the child's perceptions of sound quality as well as parents' and therapists' clinical observations. Chart data indicated that 4 of the 10 children chose to continue using a hearing aid in the contralateral ear for periods ranging from 1 month to about 18 months. In all cases, hearing aid use was discontinued due to the child's report of poor sound quality. Systematic speech perception testing was not conducted in the bimodal condition; therefore all results extracted for this study were obtained in a unilateral cochlear implant mode.

### Procedures

The clinical assessment protocol for these patients included pre-implant speech recognition testing to determine candidacy and establish baseline functioning. Post-implant testing, typically using recorded speech materials, was conducted at 6 and 12 month intervals and subsequently annually. Clinical speech recognition measures were selected on the basis of the child's linguistic abilities and ranged from parent questionnaires on auditory functioning to tests of open-set speech perception. The two most frequently administered outcome measures; the Phonetically Balanced Kindergarten Test (PBK words) and the Hearing in Noise Test for Children (HINT-C) up to 3 years post-implantation were evaluated. The PBK test is an open-set list of 50 monosyllabic words which has been extensively used in clinical speech perception assessment [20]. For this study, half-lists of 25 words were presented and the percentage of correctly identified words was documented. The HINT-C consists of 13 lists of 10 sentences with all correctly identified words tabulated to arrive at a percentage score [21]. One or two sentence lists (averaged) were administered depending on the child's attention for the test. Recorded measures of the tests were administered at 70 dB SPL (whenever possible) in a calibrated sound suite. Children were assigned a pre-implant score of 0% if the scores on other speech recognition tests indicated no open-set word recognition skills.

Pre-implant test scores were available for all but two children who could not complete open-set testing due to their limited speech recognition abilities. For the analysis, one child was assigned a score of 0% on the PBK, based on a measured score below chance on the Early Speech Perception (ESP), a pediatric closed-set test, which was administered in a monitored live voice mode. The second child was assigned a 0% score on the HINT-C test based on poor performance on the monitored live voice ESP test and a documented score of 0% for open-set words.

## Results

### Open-set words

**Figure 1:**
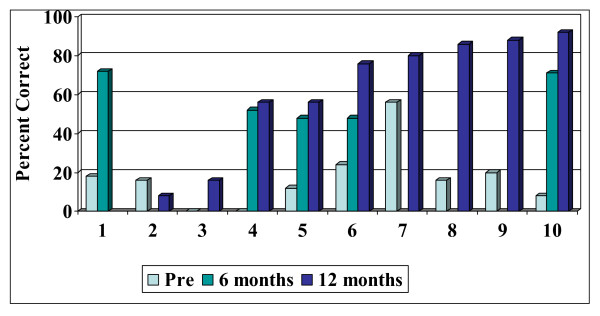
Open-set word scores.

**Figure 2:**
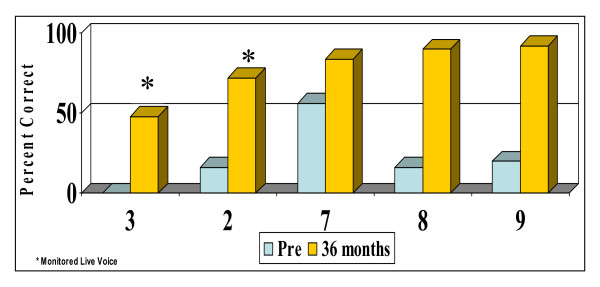
Open-set word scores (36 months post-implant).

The individual results, expressed as percent correct scores, are plotted in Figure 1 for open-set words (PBK) for the 10 patients at the 6 and 12-month follow-up intervals. One patient has 6-month follow-up data only. Long-term PBK scores are presented for 5 patients in Figure 2. The results indicate improvement in open-set auditory skills within the first 6 to 12 months of implant use for all but two children. However, the results for these two children (Patients 3 and 2, Figure 2) at the 36-month post-implant interval demonstrate that they have continued to derive substantial benefit from their implant improving from 0 and 16% on pre-implant PBK words to 48% and 72% (monitored live-voice). The majority of children showed improvement in the percentage of correct words following implantation. However,, given the extreme variability inherent to speech perception tests even in adult listeners and the potential misinterpretation of these test scores in measuring progress, we applied the binomial modelling concept described by Thornton and Raffin [22] to determine whether pre-implant and post-implant scores were significantly different for each participant. To compare scores, we used the published critical difference tables which specify the largest and smallest test score (with a 95% probability) for any given test score [22]. For example, for Patient 4, there is a 95% probability that the pre-implant score of 0% falls within the range 0%–8% and that the 12-month post-implant score of 56% lies in the range of 32%–80%, thus representing a significant difference between scores. Using the critical difference scores, 6 of the 10 children in this study showed significant improvement in open set word recognition 6 to 12 months post-implant.

Since the range of test scores does not represent a normal distribution, the group median scores are shown in Figure 4 for the pre-implant and 12 month post-implant intervals. Open-set word scores improved on average by 60%, from a pre-implant median score of 16% (range 0 to 56%) to a median score of 76% (range 8% to 92%) within 12 months of implant use.

### Open-set sentences

**Figure 3:**
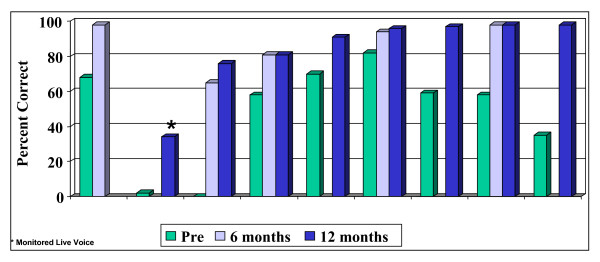
Open-set sentence scores.

A similar pattern of results is seen for open-set sentence understanding with the HINT-C sentences in quiet (Figure 3). Seven of the 9 children show considerable gains, achieving 80% or more on open-set sentence recognition within 12 months of implant experience.

**Figure 4:**
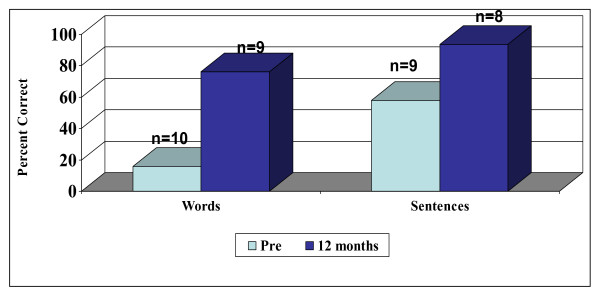
Median open-set word and sentence scores.

Figure 4 presents a summary of the group median results prior to implantation and at 12 months post-surgery. Median sentence scores improved from 58% (range 0 to 82%) to 93.5% (range 34–98%) in the first 12 months of implant experience.

## Discussion

These findings suggest rapid gains in auditory skills following cochlear implantation for 8 of the 10 patients in this study. The two children who made slower progress continue to improve their communication skills. This retrospective study cannot determine whether other child characteristics and/or family factors may have interfered with their progress.

However, as discussed by Blamey et al [14], caution must be exercised in drawing conclusions about the benefits of an intervention based on speech recognition scores as speech understanding, particularly for sentences, is not only affected by the individual's auditory capacity but also depends on linguistic abilities. As with many other published cochlear implant outcome studies, in this retrospective study, it was not possible to account for this confounding factor. Retrospective data did not allow us to quantify the linguistic abilities of these children pre and post-implant or to isolate the impact of speech and language growth over time on their speech perception scores. However, the significant improvement in auditory capacity in such a short timeframe suggests that at least some of the gains were due to the cochlear implant intervention rather than to the progress that would have occurred in the course of rehabilitation with a conventional hearing aid. These children had several years of experience with conventional acoustic amplification and auditory-verbal rehabilitation prior to cochlear implantation and were implanted based on the best clinical judgment that their auditory skills had reached a plateau with hearing aids.

Our findings are in agreement with those of Dettman et al [16] who documented significantly improved speech recognition after implantation in children with pre-implant auditory abilities outside the typical selection criteria. These results suggest that children with residual hearing may derive greater benefit from cochlear implantation than from hearing aids. The limitations of this study are those inherent to retrospective studies of small diverse clinical populations where data have been collected in a clinical context. However, the patient group in this study was quite homogeneous as all children had considerable hearing aid experience, were assessed in one clinical program and were old enough to reliably complete open-set speech recognition testing.

## Conclusion

The goal of this retrospective study was to contribute to clinical decision-making by documenting outcomes after cochlear implantation in children with residual hearing levels outside the typical audiologic criteria. A review of candidacy criteria is warranted so that children with less severe degrees of hearing loss can obtain the additional auditory stimulation (greater access to the speech spectrum) provided by a cochlear implant. Studies to date have focused primarily on auditory capacity as measured by speech recognition tests. However, to guide candidacy decision-making, it is also important to better understand the potential outcomes in children with significant residual hearing, in overall communication and academic functioning. Prospective studies and larger scale cross-sectional studies are needed to compare outcomes in multiple communication domains between children with severe hearing loss who use hearing aids and children who use cochlear implants.

## Competing interests

E. Fitzpatrick is the recipient of a doctoral fellowship from Advanced Bionics Corporation. This funding body has not been involved in any aspect of the design, data collection, or manuscript related to this study. The conclusions and opinions expressed in the manuscript are the authors' alone.

## Authors' contributions

EF was responsible for the study's conception and design, was involved in the analysis and interpretation of the data and drafted the manuscript. RM was responsible for data acquisition and participated in the analysis and interpretation of the data as well as contributed to drafting of the manuscript. DS participated in the interpretation of data, provided feedback on the intellectual content and critically reviewed the manuscript. All authors reviewed and approved the final manuscript.

## Pre-publication history

The pre-publication history for this paper can be accessed here:


